# Diverse Signals Establish the Left-Right Body Axis

**DOI:** 10.1371/journal.pbio.0020061

**Published:** 2004-02-17

Most animals (including humans) show a high level of bilateral symmetry: on the surface, the right side of our body resembles the left. A closer and deeper look, however, reveals an underlying asymmetry. The heart, for example, is on the left side in most humans, and the liver on the right. This left-right asymmetry develops early on in the embryo, and research in the past few years has revealed some of the molecular and cellular mechanisms that establish the left-right axis, which conveys positional information to cells in the growing embryo. We know that the formation of the axis relies on “crosstalk” between cells, which involves long-range signaling molecules (or ligands) and cell-surface receptors on cells that receive the signal.

The molecules involved in the formation of the left-right axis during embryogenesis, along with their functions, are conserved among vertebrates. They include members of the Transforming Growth Factor beta (TGF-ß) family—such as the agonists (or ligands) Nodal, Vg1/GDF, and activin, and the antagonist (a molecule that interferes with agonist/ligands) Lefty—on the signaling side and members of the EGF-CFC family—such as the activin receptor and its coreceptors—on the receiving side. The EGF-CFC proteins play important roles in early vertebrate embryogenesis; mutations in these genes in the zebrafish (and mouse) result in a range of developmental defects, including problems in left-right axis specification. While ligand-stimulation of the activin receptor by Nodal and Vg1/GDF requires the EGF-CFC coreceptors, activin can activate the activin pathway without a coreceptor. Lefty—being an antagonist—can block activation of the activin receptor, though it is not clear how.

Through genetic and biochemical studies in zebrafish and frog embryos, Simon Cheng, Alex Schier, and colleagues have now clarified a piece of this very complex signaling puzzle by demonstrating that Lefty inhibits a subset of TGF-ß signals—Nodal and Vg1/GDF but not activin—by blocking EGF-CFC coreceptors. They went on to show that a short, specific region of the signal molecules—accounting for less than 4% of the entire protein—determines whether the signals activate the activin receptor in an EGF-CFC coreceptor-dependent or -independent fashion and therefore governs susceptibility to Lefty. These findings suggest that subtle sequence differences between related signals can dramatically influence their function.

Gene families are thought to arise from gene duplications, and the studies described here illustrate how members of the same gene families can gain diverse roles by specific interactions with coreceptors and antagonists. Additional studies will be necessary to reveal the structural basis for the observed diversity.

**Figure:**
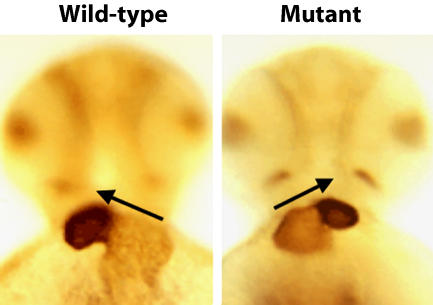
Zebrafish embryo heart loops correctly in wild-type, incorrrectly in mutant

